# Preconditioning-Activated AKT Controls Neuronal Tolerance to Ischemia through the MDM2–p53 Pathway

**DOI:** 10.3390/ijms22147275

**Published:** 2021-07-06

**Authors:** Emilia Barrio, Rebeca Vecino, Irene Sánchez-Morán, Cristina Rodríguez, Alberto Suárez-Pindado, Juan P. Bolaños, Angeles Almeida, Maria Delgado-Esteban

**Affiliations:** 1 Institute of Functional Biology and Genomics, University of Salamanca, CSIC, 37007 Salamanca, Spain; emibg7@gmail.com (E.B.); rebecavecino@usal.es (R.V.); irene_sm@usal.es (I.S.-M.); c.rodriguez@usal.es (C.R.); Alsuap77@gmail.com (A.S.-P.); jbolanos@usal.es (J.P.B.); aaparra@usal.es (A.A.); 2 Institute of Biomedical Research of Salamanca, University Hospital of Salamanca, University of Salamanca, CSIC, 37007 Salamanca, Spain; 3 Department of Biochemistry and Molecular Biology, University of Salamanca, 37007 Salamanca, Spain; 4 Centro de Investigación Biomédica en Red de Fragilidad y Envejecimiento Saludable (CIBERFES), Instituto de Salud Carlos III, 28029 Madrid, Spain

**Keywords:** AKT, MDM2, p53, PI3K, ischemic tolerance, preconditioning

## Abstract

One of the most important mechanisms of preconditioning-mediated neuroprotection is the attenuation of cell apoptosis, inducing brain tolerance after a subsequent injurious ischemia. In this context, the antiapoptotic PI3K/AKT signaling pathway plays a key role by regulating cell differentiation and survival. Active AKT is known to increase the expression of murine double minute-2 (MDM2), an E3-ubiquitin ligase that destabilizes p53 to promote the survival of cancer cells. In neurons, we recently showed that the MDM2–p53 interaction is potentiated by pharmacological preconditioning, based on subtoxic stimulation of NMDA glutamate receptor, which prevents ischemia-induced neuronal apoptosis. However, whether this mechanism contributes to the neuronal tolerance during ischemic preconditioning (IPC) is unknown. Here, we show that IPC induced PI3K-mediated phosphorylation of AKT at Ser^473^, which in turn phosphorylated MDM2 at Ser^166^. This phosphorylation triggered the nuclear stabilization of MDM2, leading to p53 destabilization, thus preventing neuronal apoptosis upon an ischemic insult. Inhibition of the PI3K/AKT pathway with wortmannin or by AKT silencing induced the accumulation of cytosolic MDM2, abrogating IPC-induced neuroprotection. Thus, IPC enhances the activation of PI3K/AKT signaling pathway and promotes neuronal tolerance by controlling the MDM2–p53 interaction. Our findings provide a new mechanistic pathway involved in IPC-induced neuroprotection via modulation of AKT signaling, suggesting that AKT is a potential therapeutic target against ischemic injury.

## 1. Introduction

In humans, the existence of transient ischemic attack (TIA) has been revealed as an endogenous preconditioned state with benefits of functional outcome in stroke patients [1,2,3]. Endogenous neuroprotection induced by a short, subtoxic ischemic stimulus, known as ischemic preconditioning (IPC), is considered a strategy in the emerging field of neuroprotection against ischemic injury [4,5,6]. Evidence shows that IPC-promoted neuroprotection depends on transcription, translation, and post-translational mechanisms, which alters the function of key proteins after ischemia [6,7,8,9,10,11]. However, the mechanism involved in IPC-induced ischemic tolerance (IT) has not been fully clarified in the human brain [12,13]. The development of new experimental approaches to understand IPC-mediated neuroprotection represents a powerful tool to decipher the endogenous mechanisms underlying brain IT [6], which have the potential to unveil novel therapeutic targets aimed at minimizing brain damage in stroke patients. 

In the last two decades, apoptotic neuronal cell death has positioned itself as an essential mechanism involved in cerebral ischemic injury [14,15,16]. In this sense, protein kinase B or AKT, a serine/threonine kinase that requires a functional phosphoinositide kinase (PI3K) to be activated, has been considered as an essential target for neuroprotective therapies after ischemia [17,18]. Recently, we demonstrated that the inhibition of the PI3K/AKT signaling pathway increases neuronal susceptibility to excitotoxicity [19]. AKT is involved in a complex anti-apoptotic signaling network [20], whose components may be present at different subcellular locations depending on tissue type [21,22]. In the heart, AKT has been involved in preconditioning-promoted cardioprotection [23]. Evidence also shows that phosphorylated AKT promotes neuronal survival in the onset of cerebral ischemia [24]. Although AKT activation may contribute to the induction of IT in the brain [25], the exact mechanism involving its IPC-mediated activation remains elusive.

In tumor cells, activation of the PI3K/AKT signaling pathway leads to MDM2 phosphorylation at Ser^166/186^, which promotes the nuclear translocation of MDM2 [26,27] and enhances its ubiquitination activity [28]. In the nucleus, MDM2 binds to p53 and promotes its ubiquitination and subsequent proteasomal degradation, which inhibits p53 function [29]. Under stress conditions, p53 could also trigger MDM2 overexpression, which conversely suppresses p53 activation in a negative feedback loop [30]. The inhibition of PI3K prevents AKT activation [31] and MDM2 phosphorylation in the preconditioned heart [32]. In this context, we previously found that in vivo brain preconditioning reduced infarct volume after transient middle cerebral artery occlusion (tMCAO) by increasing MDM2 protein level expression. Consequently, the MDM2–p53 complex attenuated ischemia-induced activation of the p53/PUMA/caspase-3 signaling pathway in primary cortical neurons [33]. Here, we dig into the role of the PI3K/AKT signaling pathway in IPC-mediated neuronal tolerance against a subsequent ischemic injury, as well as the underlying mechanism, and we mainly focus on the potential link between AKT activation and the MDM2–p53 complex.

## 2. Results

### 2.1. IPC-Promoted Neuroprotection Is Mediated by Phosphorylation of AKT at Ser^473^, Phosphorylation of MDM2 at Ser^166^, and p53 Destabilization

**Figure 1:**
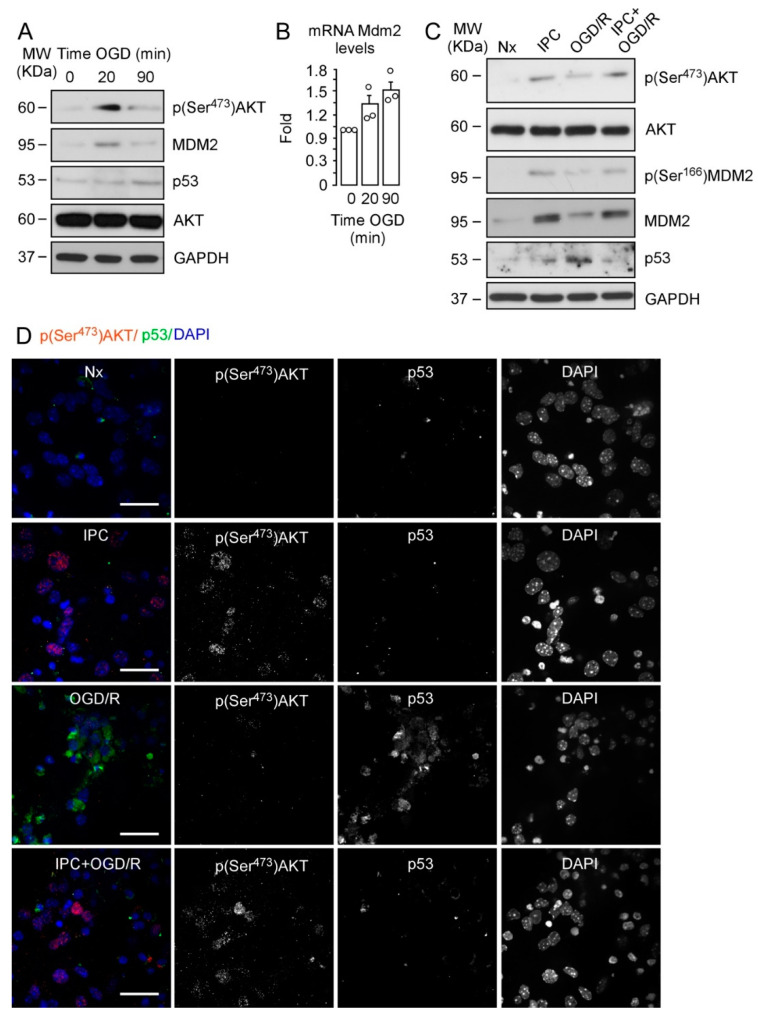
IPC promotes phosphorylation of AKT at Ser^473^ and MDM2 at Ser^166^ and reduces p53 stabilization. Mouse cortical neurons (9–10 DIV) were exposed to a validated in vitro model of IPC and ischemia, as indicated in Figure S1B. (**A**) Phosphorylation of AKT at Ser^473^, p(Ser^473^)AKT, MDM2, and p53 protein levels as detected by Western blotting after Nx and OGD (20 or 90 min). (**B**) Mdm2 mRNA levels as quantified by real-time qPCR. (**C**) AKT, p(Ser^473^)AKT, MDM2, its phosphorylated form at Ser^166^, p(Ser^166^)MDM2, and p53 were analyzed by Western blotting. AKT and GADPH were used as loading control. Relative protein abundance quantification from three or five different neuronal cultures is shown in Figure S1A,C, respectively. *M*_W_, molecular weight. (**D**) Representative images of cortical neurons stained with p(Ser^473^)AKT (red), p53 (green), and DAPI (blue, nuclear marker). Scale bar, 50 μm. Percentage of positive AKT and p53 neurons is quantified in Figure S2A.

We previously described the impact of MDM2–p53 interaction on neuronal susceptibility to ischemia [34] and IT [33]. Here, we explored the potential role of AKT on IPC-mediated neuroprotection as a candidate to be involved in the MDM2–p53 pathway. First, we confirmed that ischemia promoted AKT activation in neurons, as evidenced by AKT phosphorylation at Ser^473^. As shown in Figure 1A, short (20 min) OGD significantly induced p(Ser^473^)AKT and MDM2 expression, whereas AKT protein levels remained unaltered (Figure S1A). However, AKT phosphorylation was not observed when neurons were subjected to prolonged OGD (90 min). Moreover, MDM2 protein levels were lower, which is consistent with the higher expression of p53 protein as shown in Figure 1A.

The time-dependent upregulation of Mdm2 expression after OGD (Figure 1B) confirms that subacute ischemia may be important to induce mechanisms that prevent p53 stabilization after OGD, as previously described [33]. We used short OGD (20 min) followed by 2 h of reoxygenation as a model of IPC (Figure S1B) [33]; thus, we analyzed neuronal extracts collected at 4 h of reoxygenation after OGD (OGD/R) or after OGD preceded by the IPC protocol (IPC + OGD/R). In parallel, neurons were incubated in normoxia (Nx) or preconditioning (IPC) settings (Figure S1B). 

As shown in Figure 1C and Figure S1C, IPC induced the early activation of AKT, as revealed by phosphorylation at Ser^473^ [35], followed by MDM2 protein stabilization and phosphorylation at Ser^166^. IPC also prevented p53 stabilization induced by OGD/R (Figure 1C). Interestingly, immunofluorescence images shown in Figure 1D revealed that IPC promoted AKT phosphorylation at Ser^473^ in neurons, which predominantly accumulated in the nucleus, and decreased p53 stabilization after OGD/R (IPC + OGD/R), when compared with non-preconditioned neurons (OGD/R). Consequently, IPC prevented neuronal apoptosis and caspase-3 activation caused by OGD/R, as measured by flow cytometry (Figure S1D) and fluorimetry assays (Figure S1E), respectively. To confirm the role of p53 in IPC-mediated neuroprotection, we used neurons expressing (wild-type; wt) or not (knockout; ko) p53 protein. Our results show that neurons lacking p53 (Figure S1F) were more resistant to OGD-induced apoptosis than p53 wt neurons. Moreover, apoptosis levels in p53KO neurons were similar to those observed in preconditioned (IPC + OGD/R) wt neurons (Figure S1G), thus confirming the key role of p53 destabilization in IPC-mediated neuroprotection [33]. Our results show that IPC induced neuroprotection against an ischemic insult through a mechanism that involves phosphorylation of AKT at Ser^473^, MDM2 stabilization and phosphorylation at Ser^166^, and p53 destabilization.

### 2.2. IPC Triggers MDM2 Phosphorylation at Ser^166^ via the PI3K/AKT Pathway

**Figure 2:**
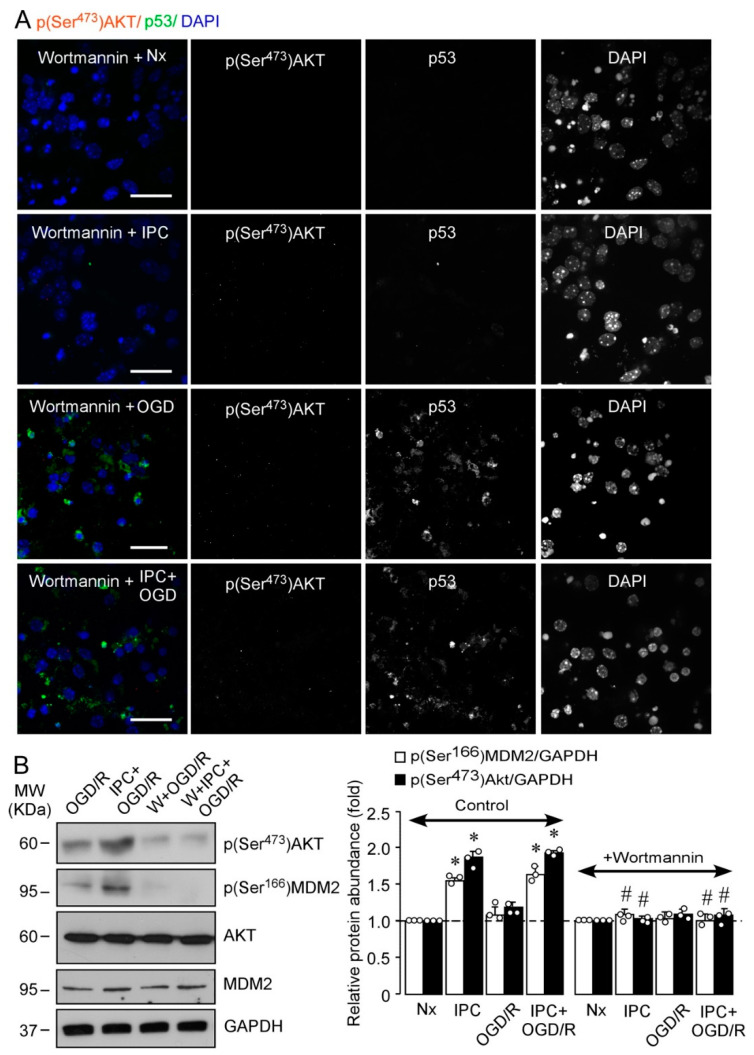
Inhibition of PI3K activity abrogates IPC-induced phosphorylation of AKT at Ser^473^ and MDM2 at Ser^166^, leading to p53 stabilization after OGD/R. Mouse cortical neurons (9–10 DIV) were treated with wortmannin for 30 min before IPC + OGD/R, Nx, IPC, and OGD/R. (**A**) Representative images of cortical neurons stained with p(Ser^473^)AKT (red), p53 (green), and DAPI (blue, nuclear marker). Scale bar, 50 μm. (**B**) p(Ser^473^)AKT and p(Ser^166^)MDM2 protein levels were analyzed by Western blot. GADPH was used as loading control. Representative blots and relative protein abundance quantification from three different neuronal cultures are shown. *M*_W_, molecular weight. Data are means ± SEM. Statistical analysis of the results was evaluated by one-way ANOVA followed by Bonferroni post hoc test. * *p* < 0.05 versus Nx and OGD/R; # *p* < 0.05 versus control.

**Figure 3:**
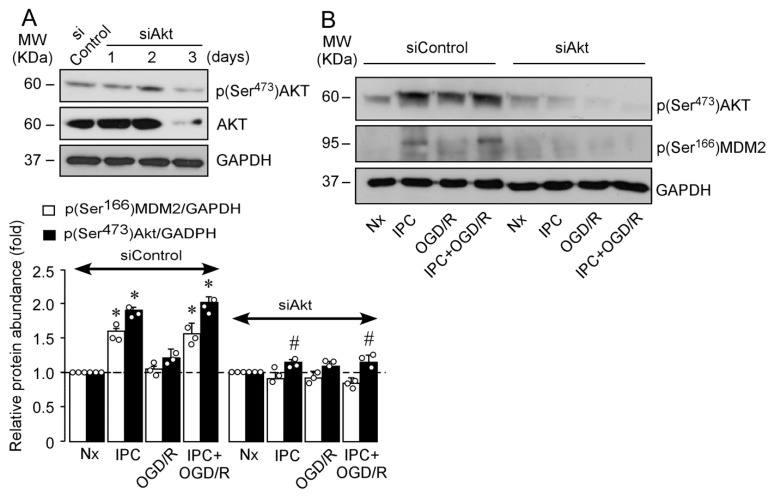
AKT downregulation prevents IPC-induced phosphorylation of AKT at Ser^473^ and MDM2 at Ser^166^ in neurons after ischemia. AKT knockdown was performed by siRNA (siAkt) transfection for (**A**) 1, 2, and 3 days in HEK-293T or (**B**) neurons at 6 DIV were transfected with siAkt for 3 days followed by Nx, IPC, OGD/R, and IPC + OGD/R protocols. p(Ser^473^)AKT, AKT, and p(Ser^166^)MDM2 protein levels were analyzed by Western Blot. GADPH was used as loading control. Representative blots are shown. Relative protein abundance quantification from three different neuronal cultures is shown. *M*_W_, molecular weight. Data are means ± SEM. Statistical analysis of the results was evaluated by one-way ANOVA followed by Bonferroni post hoc. * *p* < 0.05 versus siControl Nx and siControl OGD/R; # *p* < 0.05 versus siControl IPC and IPC+OGD/R.

The PI3K/AKT signaling pathway is involved in neuronal IT both in vitro [36] and in vivo [37]. However, the role of IPC-mediated activation of PI3K/AKT pathway in the regulation of the MDM2–p53 complex remains unexplored. In order to clarify this, neurons were incubated with the irreversible and specific inhibitor of the PI3K/AKT pathway, wortmannin [19]. As shown in Figure 2A, wortmannin abrogated IPC-enhanced (Ser^473^)AKT phosphorylation and p53 destabilization, as shown in Figure 1D. These results suggest a direct link between AKT activation and inhibition of p53-mediated neuronal apoptosis (Figure S1D) and caspase-3 activation (Figure S1E) induced after OGD/R. The main regulator of p53 stabilization, MDM2, is a target of AKT [26], which phosphorylates MDM2 at Ser^166^ and Ser^186^ [26]. PI3K inhibition with wortmannin prevented the phosphorylation of both (Ser^473^)AKT and (Ser^166^)MDM2 induced by IPC (Figure 2B). The specific AKT-mediated phosphorylation of MDM2 at Ser^166^ induced by IPC was confirmed by using a small interfering RNA (siRNA) specifically designed against AKT1 protein (siAkt), highly expressed in cortical neurons, and whose activity is essential for neuronal survival after ischemia [38]. As shown in Figure 3, siAkt reduced total AKT and p(Ser^473^)AKT protein levels at day 3 after transfection, both in HEK-293T cells (Figure 3A) and in cortical neurons (Figure 3B). Moreover, AKT knockdown (siAkt) prevented (Ser^166^)MDM2 phosphorylation (Figure 3B). These results demonstrate that the IPC-activated PI3K/AKT signaling pathway promotes MDM2 phosphorylation at Ser^166^, which may be responsible for MDM2 stabilization and consequent p53 destabilization after ischemic injury.

### 2.3. IPC-Activated AKT Triggers Nuclear MDM2 Protein Stabilization after Ischemia

**Figure 4:**
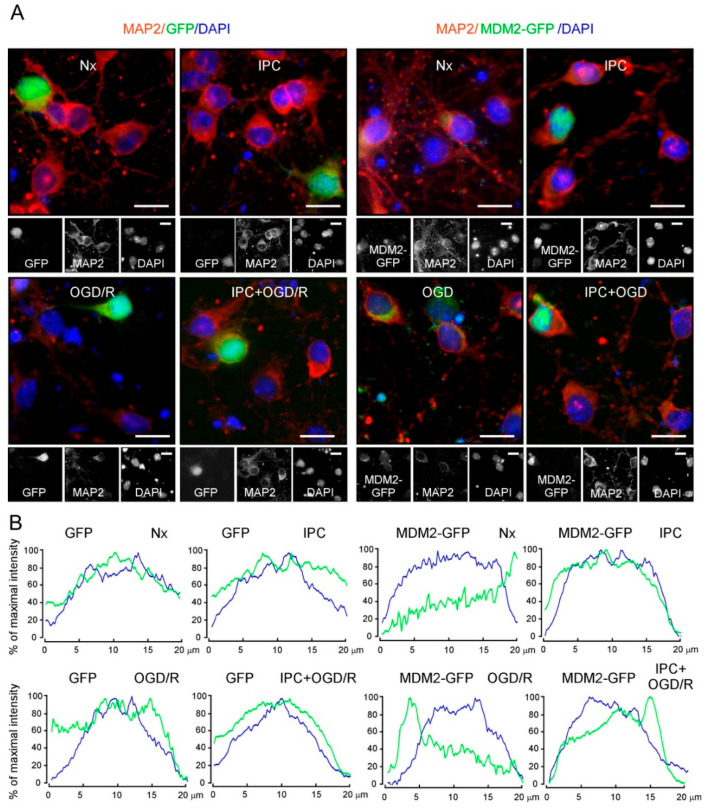
IPC triggers MDM2 nuclear stabilization in cortical neurons after ischemia. Ectopic expression of human MDM2 in neurons (9–10 DIV) is shown. GFP^+^ neurons and neurons expressing human MDM2-GFP for 24 h were subjected to Nx, IPC, OGD/R, and IPC + OGD/R conditions and were analyzed by immunofluorescence. (**A**) Representative image of cortical neurons stained with GFP (green) and MAP2 (red, neuronal marker). Scale bar, 15 μm. (**B**) Representative cross-sectional intensity profiles for GFP (green) and DAPI (blue) staining of GFP and MDM2-GFP-transfected neurons. Mean fluorescence of MDM2-GFP nuclear/cytosolic intensity ratio calculated from four different neuronal cultures is presented in Figure S2B.

The activation of AKT has been involved in nuclear translocation of MDM2 in tumor cells [26]. Considering the relevance of nuclear MDM2 stabilization for neuronal survival after ischemia [34] and, more specifically, its neuroprotective role in IPC [33], we decided to further investigate the relevance of PI3K/AKT signaling pathway in the regulation of subcellular localization of MDM2 protein. Thus, neurons or HEK-293T cells were transfected with human MDM2-tagged protein (MDM2-GFP). Representative blots of transfected HEK-293T cells and images from neurons ectopically expressing human MDM2 protein after four different experimental conditions (Nx, IPC, OGD/R, and IPC + OGD/R) are shown in Figure 4A and Figure S1H, respectively. Ectopic expression of MDM2-GFP confirmed that IPC promotes MDM2 nuclear accumulation compared with non-preconditioned ischemic (OGD/R) or normoxic (Nx) neurons (Figure 4A,B), as revealed by the quantification of nuclear/cytosolic fluorescence ratio (Figure S2B) and nuclear fluorescence intensity of MDM2-GFP (Figure S2C). 

**Figure 5:**
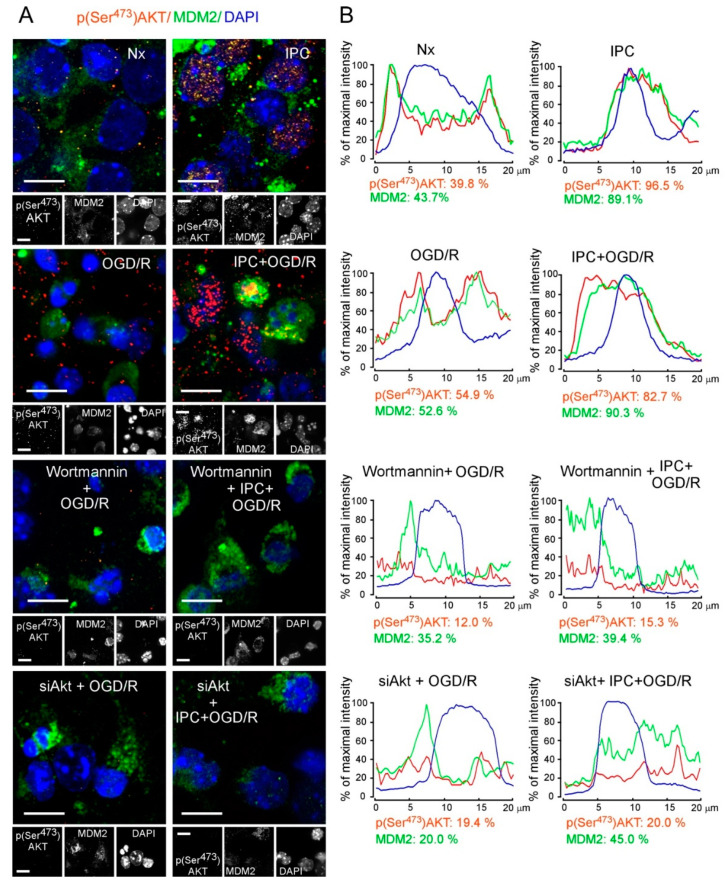
Disruption of IPC-promoted PI3K/AKT signaling pathway abrogates MDM2 nuclear translocation from the cytosol after ischemic injury. Neurons at 9–10 DIV were treated with wortmannin for 30 min or at 6 DIV were transfected with siAkt for 72 h followed by Nx, IPC, OGD/R, and IPC + OGD/R protocols. Subcellular location of MDM2 and p(Ser^473^)AKT in neurons was analyzed by immunofluorescence. (**A**) Representative images of cortical neurons untreated or treated with wortmannin or siAKT, and stained with p(Ser^473^)AKT (red), MDM2 (green), and DAPI (blue, nuclear marker). Scale bar, 10 μm. (**B**) Representative cross-sectional intensity profiles for endogenous MDM2 (green), p(Ser^473^)AKT (red), and DAPI (blue). Percentages of mean nuclear fluorescence are indicated. Maximal fluorescence intensity of p(Ser^473^)AKT and MDM2 in the nucleus of untreated neurons is calculated from four different neuronal cultures and presented in Figure S2C. In the case of neurons treated with wortmannin and siAkt, the maximal fluorescence intensity of nuclear MDM2 from five different cultures of cortical neurons is shown in Figure S2D.

Moreover, confocal immunofluorescence analysis revealed that IPC induced the colocalization of p(Ser^473^)AKT and endogenous MDM2 within the nucleus, whereas this effect was not observed under OGD/R condition (Figure 5A,B). The maximal intensity of endogenous nuclear MDM2 was higher in preconditioned (89.1% IPC and 90.3% IPC + OGD/R) than in non-preconditioned neurons (43.7% Nx and 52.6% OGD/R). Values for p(Ser^473^)AKT protein were also higher in preconditioned neurons (96.5% in IPC and 82.7% in IPC + OGD/R) compared to non-preconditioned neurons (39.8% in Nx and 54.9% in OGD/R) (Figure S2C).

Furthermore, neuron treatment with wortmannin or siAkt before the IPC or OGD/R protocol impaired MDM2 nuclear translocation after OGD/R, when compared with control neurons (Figure 5A and Figure S2D). Indeed, both treatments (wortmannin or siAkt) prevented the IPC-increased mean percentage of nuclear MDM2 maximal fluorescence intensity (39.4% and 45%) and p(Ser^473^)AKT (15.3% and 20%) after OGD/R, respectively (Figure 5B). Thus, inactivation of the AKT signaling pathway results in MDM2 protein accumulation in the cytoplasm of preconditioned neurons after ischemia. These results highlight the relevance of IPC-enhanced nuclear translocation and stabilization of MDM2 mediated by the activation of the PI3K/AKT pathway, which might confer protection against ischemic injury.

### 2.4. IPC Promotes p(Ser^473^)AKT and MDM2 Interaction, Which Enhances MDM2 Stabilization in the Nucleus and Reduces Induced Neuronal Apoptosis upon Ischemia

**Figure 6:**
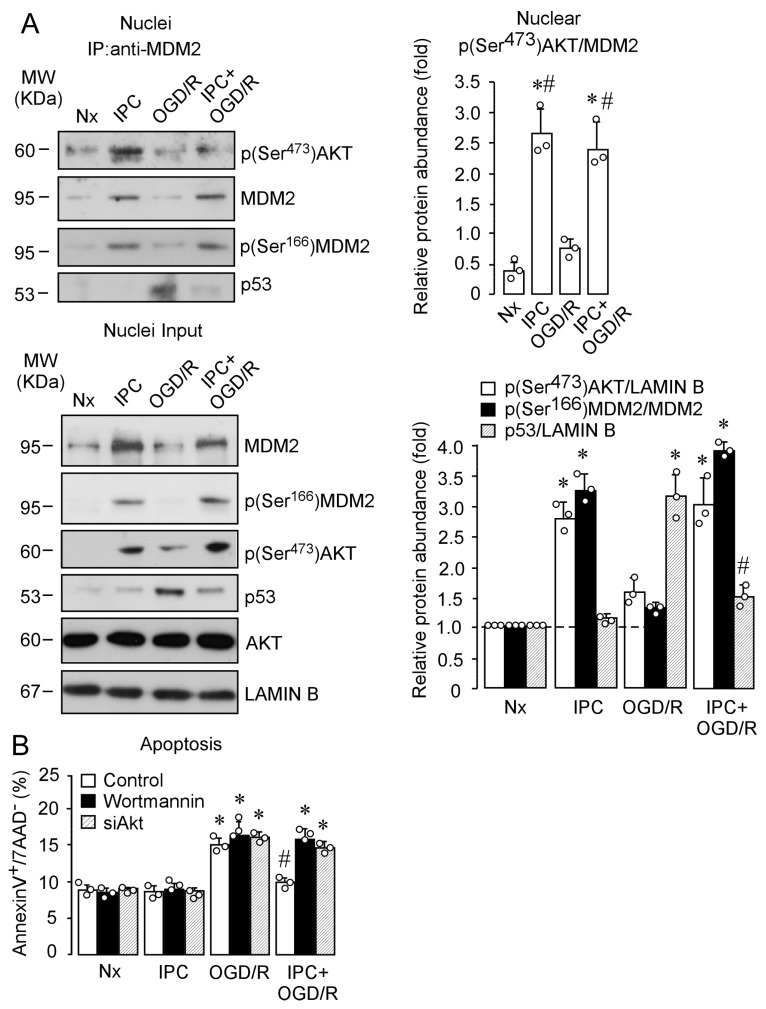
Nuclear p(Ser^4730^)AKT–MDM2 interaction may be essential to ensure IPC-promoted neuronal tolerance against ischemia. Neurons were exposed to Nx, IPC, OGD/R, and IPC + OGD/R, and the subcellular location of MDM2 and p(Ser^473^)AKT, as well as the possible protein interaction, was analyzed by nucleus–cytosol fractionation followed by coimmunoprecipitation assay. (**A**) Nuclear neuronal extracts were obtained and immunoprecipitated with anti-MDM2. Expression of MDM2 and p(Ser^473^)AKT proteins was analyzed by Western blot from neuronal immunoprecipitated (IP) samples, while 10% were loaded on SDS-PAGE as a nucleus input control. AKT and LAMIN B were used as loading control. Relative nuclear protein abundance quantification from three different neuronal cultures is presented. *M*_W_, molecular weight. (**B**) Neuronal apoptosis was analyzed by flow cytometry. Annexin V/APC-stained neurons that were 7AAD-negative were considered to be apoptotic (represented by the percentage of AnnexinV^+^/7AAD^−^ neurons). Data are means ± SEM from three different cultures of cortical neurons. In all cases, *p* < 0.05 was considered significant. Statistical analysis of the results was evaluated in Figure 6A by Student’s *t*-test. * *p* < 0.05 versus Nx; # *p* < 0.05 versus OGD/R. Statistical analysis of the results was evaluated in Figure 6B by one-way ANOVA followed by Bonferroni post hoc. * *p* < 0.05 versus control Nx; # *p* < 0.05 versus control OGD/R.

Following demonstration of the role of IPC-enhanced activation of PI3K/AKT pathway in the nuclear stabilization of MDM2, we further investigated whether p(Ser^473^)AKT and MDM2 interacted within the nucleus (Figure 6A). MDM2 immunoprecipitation from nuclear protein extracts, followed by immunoblotting against MDM2 and p(Ser^473^)AKT, revealed that IPC promoted the interaction between p(Ser^473^)AKT and MDM2, after OGD/R, thus preventing OGD/R-induced nuclear p53 stabilization, as shown in the nucleus input (Figure 6A). 

Lastly, we studied the detrimental effect of PI3K/AKT pathway disruption on neuronal apoptosis (Figure 6B). AKT inhibition counteracted the protective effect of IPC prior to OGD, which confirms the neuroprotective role of AKT–MDM2 in the context of IT. Our results, thus, demonstrate that IPC induces phosphorylation and activation of AKT, which promotes MDM2 phosphorylation at Ser^166^ and nuclear translocation, where it interacts with p(Ser^473^)AKT. This mechanism may contribute to enhanced nuclear stabilization of MDM2, which plays an essential role in IPC-induced ischemic tolerance.

## 3. Discussion

Our results reveal that IPC-mediated activation of the PI3K/AKT signaling pathway triggers neuronal IT by controlling the MDM2–p53 complex in primary cortical neurons. We first confirmed the efficiency of the preconditioning in terms of neuroprotection [33,39,40,41] using a validated IPC experimental model. We found that experimental IPC induced by a short (20 min) oxygen and glucose deprivation (OGD) followed by 2 h of reoxygenation resulted in neuroprotection, as shown by the prevention of both neuronal apoptosis and caspase-3 activation induced by prolonged OGD (90 min) followed by 4 h of reoxygenation (OGD/R). We show that IPC reduces caspase-3 activation in cortical neurons, which correlates with less apoptosis after a subsequent and more severe ischemic insult. 

The balance between pro- and antiapoptotic signals is fundamental to ensure neuronal survival after ischemia [3,33,38,42,43,44]. Although the relevance of such events has been shown in both hemorrhagic and ischemic in vivo stroke models [43,45], the mechanisms that regulate these signaling pathways are not yet fully understood in the context of ischemic tolerance. 

The role of antiapoptotic AKT and its related pathways have been extensively studied in cancer cells [46,47] and brain tissue [38,48]; however, so far, the role of the AKT/MDM2–p53 signaling pathway in IPC-mediated neuronal tolerance against ischemic injury remains elusive. Here, we found that the activation of the PI3K/AKT signaling pathway caused by IPC promotes phosphorylation of MDM2 at Ser^166^, which triggers its nuclear translocation and protein stabilization, preventing p53-induced apoptosis via caspase-3 activation after ischemia.

The activation of AKT via phosphorylation promotes neuronal survival [24,49] and may contribute to the induction of IT [25,50]. Our results show that the relative abundance of AKT protein is unchanged under ischemic or preconditioning stimuli. Interestingly, we found that early PI3K-mediated phosphorylation of AKT at Ser^473^ prevents ischemia-induced p53 stabilization in the preconditioned neurons. The effect was not due to modifications in p53 mRNA levels [33,34,44], but to decreased p53 protein levels due to IPC prior OGD/R. Since MDM2 is the main regulator of p53 stabilization and is also a direct target of AKT, our results point out the role of the AKT/MDM2–p53 signaling pathway in neuronal tolerance to ischemia. 

MDM2 mRNA rapidly increases after OGD [34], but MDM2 activity is mainly controlled by post-translational modifications, particularly phosphorylation [51]. In good agreement with this, we found that, once activated by phosphorylation after IPC, AKT in turn phosphorylates MDM2 at residue Ser^166^, which is located in close proximity to the nuclear localization signal [52], and this effect is maintained after OGD/R injury. In fact, our results show that phosphorylation of MDM2 at Ser^166^ is sufficient to exert the IPC-mediated neuroprotective effect via p53 destabilization. Thus, herein, we identified a time-dependent activation of AKT/MDM2–p53 pathway after ischemic injury and, indeed, we demonstrate that IPC-activated AKT triggered nuclear translocation of ectopic MDM2, as well as endogenous protein stabilization. Moreover, AKT remains active within the nucleus, where PI3K could also migrate in response to oxidative stress and then account for AKT phosphorylation [53]. The inhibition of PI3K-mediated phosphorylation of AKT or AKT knockdown promotes the retention of MDM2 protein in the cytoplasm, and it prevents Ser^166^ phosphorylation of MDM2, as well as IPC-mediated neuroprotection against ischemia-induced neuronal apoptosis. On the contrary, we showed that active AKT binds to nuclear MDM2 protein. As a consequence, active AKT promotes both phosphorylation of MDM2 and its nuclear stabilization, which contribute to IPC-mediated neuroprotection. Our results reveal that IPC-promoted neuroprotection was dependent on PI3K-mediated AKT activation, which phosphorylated MDM2 at Ser^166^, promoting MDM2 nuclear accumulation after an ischemic insult. Accordingly, inhibition of PI3K/AKT by wortmannin or AKT depletion by siRNA abolished IPC-promoted neuroprotection, leading to p53 stabilization and the subsequent neuronal apoptosis after ischemia. Hence, our results help to clarify the essential role of IPC-dependent activation of the AKT–MDM2 pathway in neuronal survival against ischemic injury. 

The p53 protein is involved in the control of neuronal death/survival determining prognosis in stroke patients [34,42,54], as well as in TIA patients [3]. In fact, p53 stabilization compromises preconditioning-mediated neuroprotection to ischemia/reperfusion injury [33]. The MDM2–p53 interaction will, therefore, be critical for neuronal survival in this context [34] and for IPC-mediated tolerance against ischemic injury [33]. Thus, the control of such interaction will also have an impact on stroke outcome. In this context, we recently found that a single-nucleotide polymorphism (SNP) 309T>G in the MDM2 promoter determines the expression of MDM2 and, in turn, modulates the recovery of patients suffering from stroke [34]. Additionally, we observed that a *Tp53* gene SNP (rs1042522) modulates mitochondrial p53 stabilization and neuronal tolerance to ischemia, while predicting the functional recovery of patients who suffer a TIA prior to stroke [3]. Therefore, the control of p53 apoptotic pathways will be essential to ensure the neuroprotective effect of IPC. These results provide a translational approach to the study that could be implemented in the future for the benefit of patients, and they pose PI3K/AKT–MDM2–p53 signaling pathway as an essential target for the preconditioning-promoted IT strategies in ischemic stroke. 

In summary, we demonstrate that IPC-enhanced PI3K/AKT signaling pathway promotes phosphorylation of MDM2 at Ser^166^, leading to MDM2 nuclear translocation and its stabilization, which triggers neuronal IT by promoting p53 destabilization and subsequent inactivation of apoptotic death induced after ischemic insult. Our results highlight the potential benefits of early activation of AKT in IPC-mediated neuronal tolerance, which regulates the MDM2–p53 apoptotic pathway under ischemic injury. These findings highlight an opportunity to understand the mechanisms that regulate neuronal the AKT–MDM2–p53 signaling pathway to develop novel neuroprotective strategies for IT-related disorders.

## 4. Materials and Methods

### 4.1. Primary Cultures of Cortical Neurons 

Neuronal cultures were prepared from C57Bl/6J or p53-null (Tp53^−/−^, B6.129S2, The Jackson Laboratory) mouse embryo (14.5E) cortices. Neurons were seeded at 1.8 × 10^5^ cells/cm^2^ in Neurobasal medium supplemented with 2% B27 and 2 mM glutamine (Invitrogen, Madrid, Spain) and incubated at 37 °C in a humidified 5% CO_2_-containing atmosphere [55].

### 4.2. Oxygen Glucose Deprivation and Preconditioning Models

After 9–10 days in vitro (DIV), neurons were exposed to oxygen and glucose deprivation (OGD) by incubating cells at 37 °C for 90 min in an incubator equipped with an airlock and continuously gassed with 95% N_2_/5% CO_2_. The incubation medium (buffered Hanks’ solution without glucose: 5.26 mM KCl, 0.43 mM KH_2_PO_4_, 132.4 mM NaCl, 4.09 mM NaHCO_3_, 0.33 mM Na_2_HPO_4_, 2 mM CaCl_2_, and 20 mM HEPES, pH 7.4) was previously gassed with 95% N_2_/5% CO_2_ for 30 min. Under these conditions, oxygen concentrations in the incubation medium were 6.7 ± 0.5 μM as measured with a Clark-type oxygen electrode [56,57]. When indicated, the neurons were exposed to ischemic preconditioning (IPC; short OGD for 20 min followed by 2 h of reoxygenation) prior to a subsequent prolonged ischemia (OGD, 90 min) and 4 h of reoxygenation (IPC + OGD/R) (Figure S1B). In parallel, neurons were incubated in normoxia (Nx) at 37 °C in a humidified atmosphere of 95% air/5% CO_2_ or ischemic preconditioning (IPC). When indicated, neurons were incubated 30 min before IPC in buffered Hanks’ solution (pH 7.4), in the absence or presence of wortmannin (100 nmol/L), as described previously [19].

### 4.3. Cell Transfections

Neurons (8 DIV) or HEK-293T cells were transfected with a plasmid vector expressing YFP tagged Mdm2 from MDM2 human promoter. MDM2p/Mdm2-YFP was a gift from Uri Alon & Galit Lahav (Addgene plasmid # 53962, Watertown, MA, USA) [58]. When required, an empty vector (pYFP) was used as control in the same conditions. Plasmid transfection was performed using Lipofectamine^®^ LTX (Invitrogen, Carlsbad, MA, USA), according to the manufacturer’s instructions. Cells were transfected with 1.5 µg/µL of the plasmid vectors and used after 24 h. AKT knockdown in 6 DIV neurons was achieved by transfection with small interfering double-stranded ribonucleotides (siRNA). Targeted sequences were as follows: 5′–CUCAAGUACUCAUUCCAGAtt–3′, antisense: 5′–UCUGGAAUGAGUACUUGAGgg–3′(mouse, s62216, corresponding to nucleotides 1006–1025, GenBank accession number NM_009652) [59]. As a negative control, we used Silencer™ Select Negative Control No. 1 siRNA (siControl). All siRNAs were purchased from Ambion^®^, Invitrogen^®^, Thermo Fischer Scientific (Offenbach, Germany). According to the degree of protein knockdown, the efficiency of transfection of siRNA was estimated to be 70–80% at 3 days post transfection. For silencing experiments, neurons were transfected with siRNA (10 nM) using Lipofectamine^®^ RNAiMAX (Invitrogen), following the manufacturer’s instructions. Neurons were further incubated in Neurobasal medium for 72 h before their use.

### 4.4. Flow Cytometric Detection of Apoptotic Cell Death 

Neurons were carefully detached from the plates using 1 mM EDTA tetrasodium salt in PBS (pH 7.4) and were stained with annexin V/APC and 7-AAD, performed exactly as previously described [55]. 

### 4.5. Caspase-3 Activity Assay

Caspase-3 activity was assessed in cell lysates [33] and according to the manufacturer’s instructions using the Fluorimetric Assay kit CASP3F from SIGMA and read at emission at a wavelength of 405 nm. The method is based on the release of the fluorescent 7-amino-4-methyl coumarin (AMC) moiety. The AMC concentration is calculated using an AMC standard.

### 4.6. Immunoblots and Co-Immunoprecipitation Assay

Neurons were lysed in buffer containing 1% SDS, 2 mM EDTA, 150 mM NaCl, 12.5 mM Na_2_HPO_4_, and 1% Triton X-100 (NP40: 1% NP40, EDTA diK^+^ 5 mM, Tris pH8 20 mM, NaCl 135 mM, and 10% glycerol) supplemented with phosphatase inhibitors (1 mM Na_3_VO_4_ and 50 mM NaF) and protease inhibitors (100 mM phenylmethylsulfonyl fluoride, 50 µg/mL anti-papain, 50 µg/mL pepstatin, 50 µg/mL amastatin, 50 µg/mL leupeptin, 50 µg/mL bestatin, and 50 µg/mL soybean trypsin inhibitor), stored on ice for 30 min and boiled for 5 min. Aliquots of lysed extracts were subjected to SDS polyacrylamide gel (MiniProtean^®^, Bio-Rad) and blotted with antibodies overnight at 4 °C. Antibodies used were anti-AKT (9272), anti-p(Ser^473^)AKT (9271), anti-cleaved caspase-3 (Asp175, 9661) (Cell Signaling, Danvers, MA, USA), anti-p53 (554157, BD Biosciences), anti-MDM2 (2A10, ab-16895), anti-p(Ser^166^)MDM2 (ab131355), anti-GFP (ab290; also detects YFP) (Abcam, Cambridge, UK), anti-LAMIN B (sc-374015, Santa Cruz Biotechnology, Heidelberg, Germany), and anti-GAPDH (Ambion, Cambridge, UK) overnight at 4 °C. After incubation with horseradish peroxidase-conjugated goat anti-rabbit IgG (Pierce, Thermo Scientific) or goat anti-mouse IgG (Bio-Rad), membranes were immediately incubated with enhanced chemiluminescence SuperSignal West Dura (Pierce) for 5 min before exposure to Kodak XAR-5 film for 1 to 5 min and the autoradiograms were scanned. Band intensities were quantified using ImageJ 1.48v software, as described previously [60]. For the co-immunoprecipitation assay, neurons were lysed in ice-cold buffer containing 50 mM Tris (pH 7.5), 150 mM NaCl, 2 mM EDTA, 1% NP-40) supplemented with phosphatase inhibitors described above. After clearing debris by centrifugation, neuronal lysates (100 mg) were incubated with 1 mg of the antibody for 24 h at 4 °C followed by the addition of 10 mL of protein A-agarose (GE Healthcare Life Sciences) for 2 h at 4 °C. Immunoprecipitates were extensively washed with lysis buffer and resolved by SDS-PAGE and immunoblotted with indicated antibodies [61]. The relative protein abundances are shown in Figure S1. Full blots and gel scans are included in Figure S3.

### 4.7. Immunocytochemistry and Image Analysis 

Neurons were grown on glass coverslips and fixed with 4% (*w*/*v*, in PBS) paraformaldehyde for 30 min and immunostained with rabbit anti-phosphoAKT (Ser^473^; 9271; Cell Signaling, MA, USA), mouse anti-MDM2 (2A10, ab-16895), mouse anti-MAP2 (1:500; M#1406, Sigma-Aldrich, St. Louis, MO, USA) [55], mouse anti-p53 (1:200; 554157, BD Pharmingen, San Diego, CA, USA), and anti-GFP (1:1000; ab290; also validated to detect YFP). Immunolabeling was detected using secondary antibodies anti-rabbit IgG–Cy3 or anti-mouse IgG–Cy2 (1:500; Jackson ImmunoResearch. Cambridge, UK). Nuclei were stained with 4′,6-diamidino-2 phenylindole (DAPI; D9542, Sigma-Aldrich). Coverslips were washed, mounted in SlowFade light antifade reagent (Invitrogen) on glass slides, and examined using a microscope (Nikon Inverted microscope Eclipse Ti-E, (NY, USA) equipped with 40× objective, a pre-centered fiber illuminator Nikon Intensilight C-HGFI, and a black-and-white charge-coupled device digital camera Hamamatsu ORCAER or a scanning laser confocal microscope (“Spinning Disk” Roper Scientific Olympus IX81, Tokyo, Japan) with three lasers 405, 491, and 561 nm, equipped with 40×, 63×, and 100× PL Apo oil-immersion objective for high-resolution imaging and device digital camera Evolve Photometrics. All microscope settings were set to collect fluorescent images below saturation and were kept constant for all images taken in the experiment. Images were analyzed with the ImageJ 1.48v software (National Institutes of Health). The percentage of p(Ser^473^)AKT^+^ and p53^+^ neurons and the quantification of maximal protein fluorescence intensity of p(Ser^473^)AKT and p53 are shown in Figure S2A. In MDM2-GFP-transfected neurons, the nucleocytoplasmic distribution of MDM2-GFP was calculated as the ratio of the nuclear mean fluorescence to the cytoplasmic mean fluorescence of endogenous MDM2, measured in 24 neurons (six neurons per condition in four different neuronal cultures) (Figure S2B) [62]. To quantify the maximal nuclear fluorescence intensity of endogenous MDM2 staining and pSer^473^AKT, 40 neurons (10 neurons per condition in four different cultures) were measured (Figure S2C), as described previously [44]. In the representative cross-sectional intensity profiles shown in Figure 5B, the percentage of p(Ser^473^)AKT and MDM2 indicated below each condition was quantified as nuclear mean fluorescence. In all cases, nuclei were identified by DAPI staining. The maximal nuclear MDM2 fluorescence intensity in neurons treated with wortmannin or siAkt is shown in Figure S2D.

### 4.8. Statistical Analysis 

Experimental results were evaluated by one-way analysis of variance, followed by the Bonferroni post hoc test, used to compare values between multiple groups. The results are expressed as means ± SEM. Student’s *t*-test was used for comparisons between two groups of values. In all cases, *p* < 0.05 was considered significant (* *p* < 0.05 versus Nx; # *p* <0.05 versus OGD). Statistical analyses were performed using SPSS Statistics 24.0 for Macintosh (IBM). 

## Supplementary Materials

The following are available online at https://www.mdpi.com/article/10.3390/ijms22147275/s1.
