# Supplementary material for: Preconditioning-Activated AKT Controls Neuronal Tolerance to Ischemia through the MDM2–p53 Pathway
Source: Int J Mol Sci. 2021 Jul 6;22(14):7275. doi: 10.3390/ijms22147275 (PMC8304232; doi:10.3390/ijms22147275)
Supplement: Supplementary file 1 [file ijms-22-07275-s001.zip › ijms-1268927-supplementary.pdf]

Supplementary Figures

Title: **Preconditioning-activated AKT controls neuronal tolerance to ischemia through the MDM2-p53 pathway**

Authors: Barrio E[1, †], Vecino R[1,2, †], Sanchez-Moran I[1], Rodriguez, C[1,2,3], Suarez-Pindado A[1], Bolaños JP[1,2,3,4], Almeida A[1,2,3] and Delgado-Esteban M[1,2,3,*]

[1]Institute of Functional Biology and Genomics, University of Salamanca, CSIC, Salamanca, Spain

[2]Institute of Biomedical Research of Salamanca, University Hospital of Salamanca, University of Salamanca, CSIC, Salamanca, Spain

[3]Department of Biochemistry and Molecular Biology, University of Salamanca, Spain

[4]Centro de Investigación Biomédica en Red de Fragilidad y Envejecimiento Saludable (CIBERFES), Instituto de Salud Carlos III, Madrid, Spain

[†]Equally contributed to this work

*Corresponding author:
María Delgado-Esteban
Institute of Biomedical Research of Salamanca
University Hospital of Salamanca-University of Salamanca
Calle Zacarías González 2,
37007 Salamanca, Spain
Tel. +34923294908 (5453)
Fax. +34923224876
E-mail: mdesteban@usal.es

ORCID: 000-0002-6205-6611

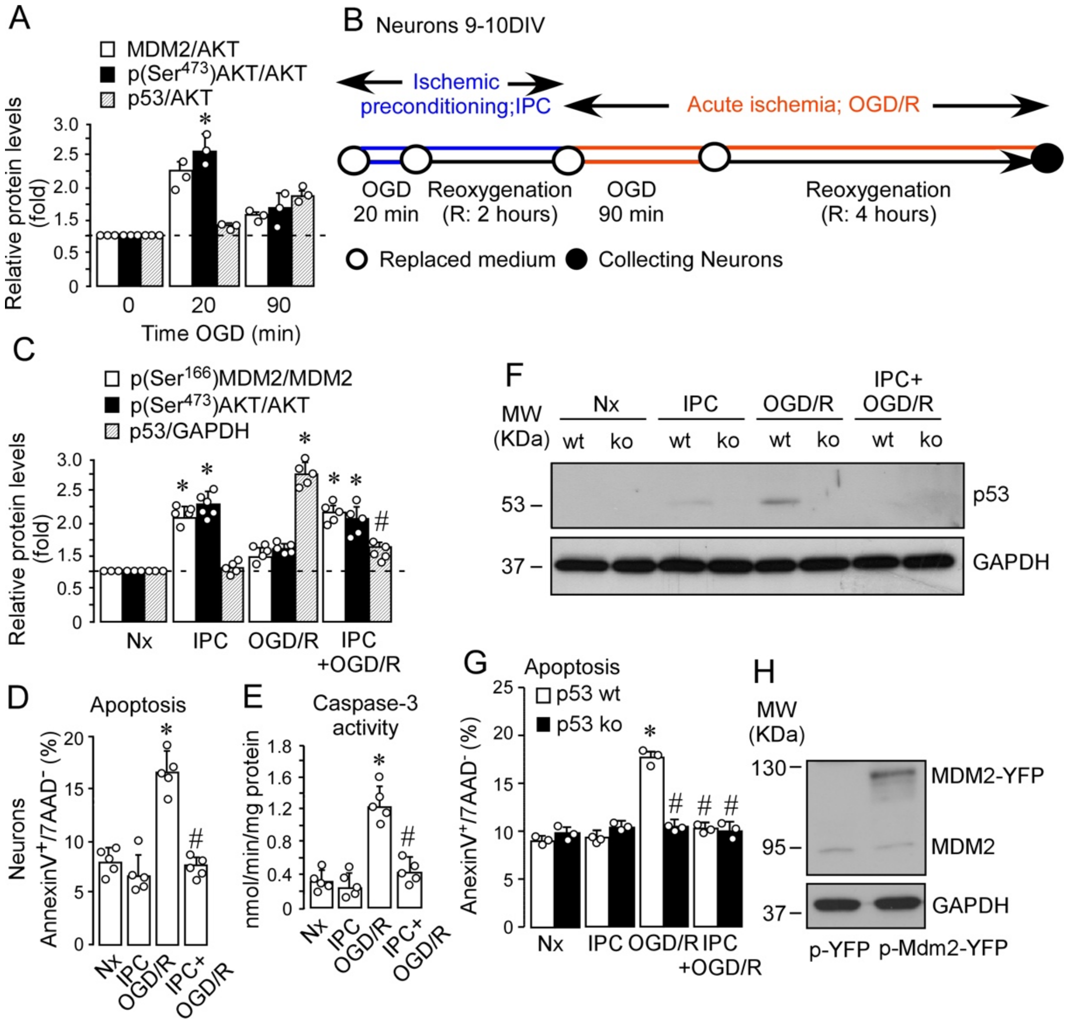

Figure S1. IPC Prevents Ischemia-Induced p53 Stabilization and Neuronal Apoptosis. (**A**) Relative protein abundance quantification of MDM2, p(Ser^{473})AKT, p(Ser^{166})MDM2 and p53 including blots shown in Figure 1A. AKT and GAPDH were used as loading control. (**B**) Schematic representation of the *in vitro* model of IPC (20 minutes of OGD followed by 2 hours of reoxygenation) and 4 hours of reoxygenation after 90 minutes of OGD (OGD/R) in primary cultures of cortical neurons. (**C**) Relative protein abundance quantification of MDM2, p(Ser^{473})AKT, p(Ser^{166})MDM2 and p53 including blots shown in Figure 1C. Analysis of apoptotic death, by (**D**) flow cytometry (percentage of AnnexinV^{+}/7AAD^{-} neurons) and (**E**) measurement of caspase-3 activity by fluorimetry assay, after four conditions Nx, IPC, OGD/R and IPC + OGD/R. (**F**) Representative blot of p53 expression or absence of the protein in wild type (wt) or knockout (ko) neurons, respectively, after Nx, IPC, OGD/R and IPC + OGD/R protocols. (**G**) Apoptosis analysis by flow cytometry assay in p53 wt or ko neurons after four indicated conditions. (**H**) Representative blot from transfected HEK-293T with p-Mdm2-YFP or empty p-YFP. In all cases, data are means ± S.E.M. from three to five different cultures of cortical neurons. Blots were quantified using the ImageJ 1.48v software (National Institutes of Health). Statistical analysis was evaluated by Student's t-test in Figures S1A,C-E and by one-way ANOVA followed by Bonferroni post hoc in Figure S1G. $p < 0.05$ was considered significant. * $p < 0.05$ versus Nx. # $p < 0.05$ versus OGD/R.

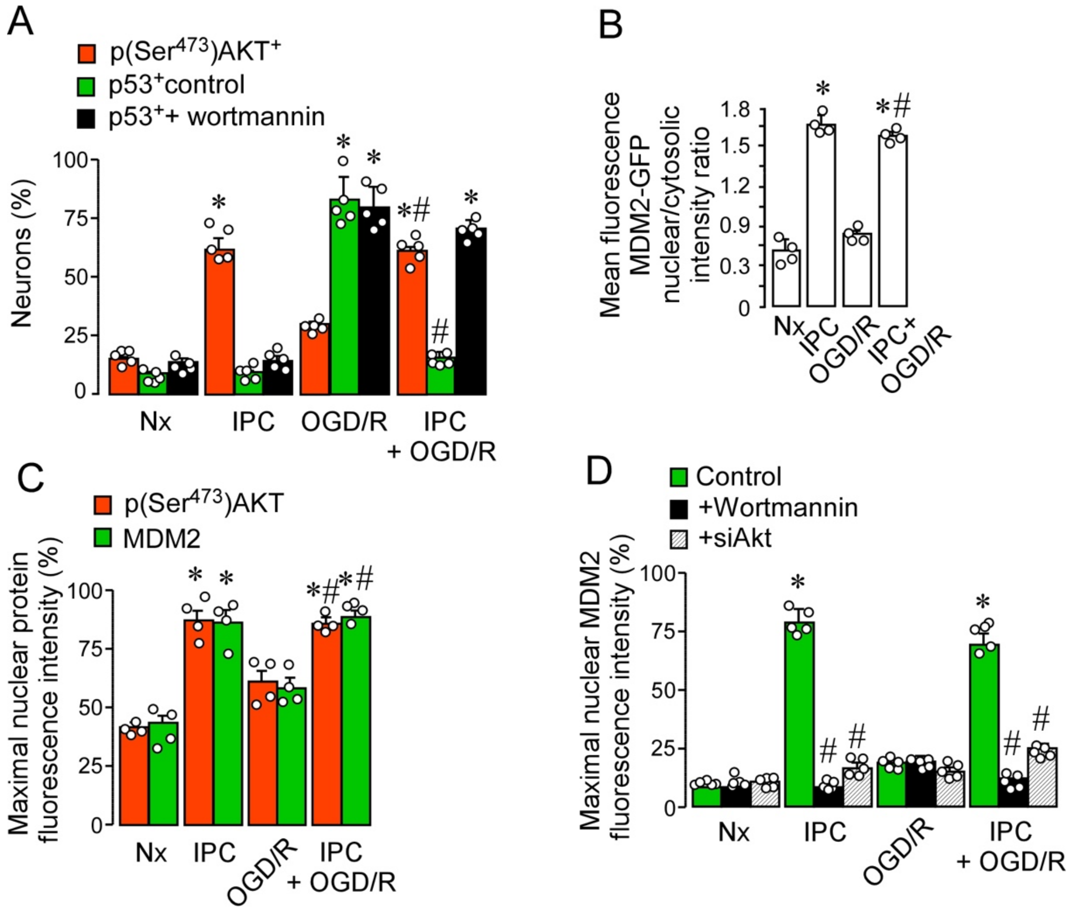

Figure S2. Quantification of the Fluorescence Intensity from Fluorescence Images. (**A**) Percentage of neurons expressing p(Ser^{473})AKT and p53, including images shown in Figures 1D and 2A. (**B**) Quantification of mean fluorescence intensity of MDM2-GFP nuclear/cytosolic ratio in transfected neurons from four different neuronal cultures and images shown in Figure 4A. (**C**) Quantification of maximal fluorescence intensity of p(Ser^{473})AKT and endogenous MDM2 in cortical neurons from four different neuronal cultures, including images shown in Figure 5A. (**D**) Maximal fluorescence intensity of nuclear MDM2 in neurons treated with wortmannin and transfected with siAkt or not (control) included from images shown in Figure 5A. Images were quantified using the ImageJ 1.48v software (National Institutes of Health). In Figures S2 A-C, data are means ± S.E.M. from four to five different cultures of cortical neurons. Student's t-test was used for comparisons between two groups of values. $p < 0.05$ was considered significant. *$p<0.05$ versus Nx. # $p < 0.05$ versus OGD/R. In Figure S2D *$p < 0.05$ versus Nx or OGD/R control. # $p < 0.05$ versus IPC or IPC + OGD/R (one-way ANOVA followed by the Bonferroni post hoc test).

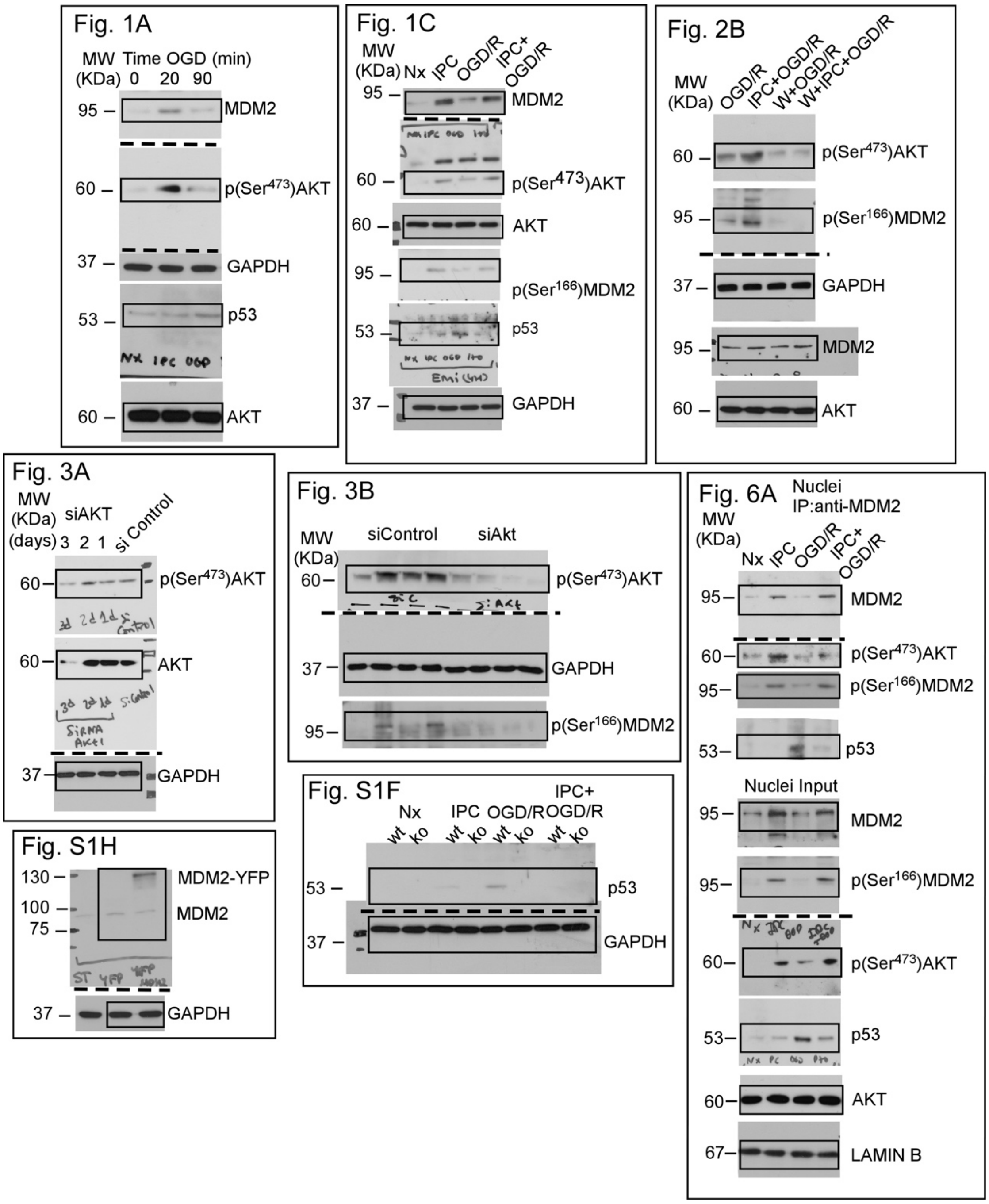

Figure S3. Representative Full Images from Blots Shown in Figures 1A, 1C, 2B, 3A, 3B, 6A and Supplementary images from figures S1F and S1H. Boxed areas were cropped for designated figures. Dotted lines denote separations of membranes for dual labeling.
